# Life-course trajectories of working conditions and successful ageing

**DOI:** 10.1177/14034948211013279

**Published:** 2021-05-25

**Authors:** Charlotta Nilsen, Alexander Darin-Mattsson, Martin Hyde, Jonas W. Wastesson

**Affiliations:** 1 Aging Research Center (ARC), Karolinska Institutet/Stockholm University, Stockholm, Sweden; 2 Stress Research Institute, Stockholm University, Stockholm, Sweden; 3 Institute of Gerontology, Jönköping University, Jönköping, Sweden; 4 Centre for Innovative Ageing, Swansea University, Swansea, UK; 5 Department of Medical Epidemiology and Biostatistics, Karolinska Institutet, Stockholm, Sweden

**Keywords:** Work-related stress, substantive complexity, physical working conditions, accumulation, de-accumulation, successful ageing, longitudinal

## Abstract

**Aims:**

As populations are ageing worldwide, it is important to identify strategies to promote successful ageing. We investigate how working conditions throughout working life are associated with successful ageing in later life.

**Methods:**

Data from two nationally representative longitudinal Swedish surveys were linked (*n*=674). In 1991, respondents were asked about their first occupation, occupations at ages 25, 30, 35, 40, 45 and 50 years and their last recorded occupation. Occupations were matched with job exposure matrices to measure working conditions at each of these time points. Random effects growth curve models were used to calculate intra-individual trajectories of working conditions. Successful ageing, operationalised using an index including social and leisure activity, cognitive and physical function and the absence of diseases, was measured at follow-up in 2014 (age 70 years and older). Multivariable ordered logistic regressions were used to assess the association between trajectories of working conditions and successful ageing.

**Results:**

Intellectually stimulating work; that is, substantive complexity, in the beginning of one’s career followed by an accumulation of more intellectually stimulating work throughout working life was associated with higher levels of successful ageing. In contrast, a history of stressful, hazardous or physically demanding work was associated with lower levels of successful ageing.

**Conclusions:**

**Promoting a healthy workplace, by supporting intellectually stimulating work and reducing physically demanding and stressful jobs, may contribute to successful ageing after retirement. In particular, it appears that interventions early in one’s employment career could have positive, long-term effects.**

## Background

As populations are ageing, it is important to promote health in later life. Working conditions play an important role in shaping health [1–4]. However, evidence is unclear as to whether work has positive or negative impacts on health and wellbeing in later life [3–6]. We will address this by investigating how the accumulation of physical and psychosocial working conditions over the life course relates to successful ageing in older adults.

The long-term impact of psychosocial working conditions on cognitive function later in life is well documented. Intellectually stimulating jobs have a protective effect on cognitive function and dementia at older ages [3, 4, 7]. In contrast, stressful jobs are linked with cognitive decline and dementia [7, 8]. Growing evidence shows that job strain is associated with limitations in physical function later in life [5, 9]. However, existing studies tend to use relatively crude measures of working conditions (such as one specific type of working condition) or static measures (e.g. main lifetime employment assessed once) [3, 4, 9, 10], and usually focus on one or two discreet outcomes later in life [3–5, 8–10]. Such approaches may underestimate long-term consequences of working life on health in later life [5]. To redress this, we model the accumulation and de-accumulation of diffe-rent working conditions throughout working life. Following Ferraro and Morton [11] we define accumulation as a process of amassing one or more things, whether desirable or undesirable, within domains of interest. In this paper the phenomena of interest are various types of occupational characteristics; that is, whether jobs are complex (i.e. intellectually stimulating), hazardous, physically demanding or stressful. Moreover, Ferraro and Morton note that there is a relative lack of research on the ‘accumulation of advantage’ and ‘de-accumulation’. We address both in this paper by looking at both increasing and decreasing trajectories of both poor working conditions; for example, hazardous jobs and good working conditions; that is, intellectually stimulating jobs. Another limitation of much of the existing research is the focus on single health outcomes. This ignores the complexity of health and wellbeing in later life. In contrast we use a multidimensional measure of successful ageing. Although there is not an agreed upon definition of successful ageing, the consensus is that it is ageing without chronic diseases while maintaining high cognitive and physical function [12, 13]. In contrast to ‘normal ageing’ it is presented as what may be possible if people are able to maintain healthy lifestyles and reserve capacities over the life course [14].

We hypothesise that those who start with poor working conditions and experience an accumulation of disadvantage will be least likely to age successfully, and those who start with good working conditions and experience an accumulation of advantage will be most likely to age successfully.

## Methods

### Data

Data from the Level of Living Survey (LNU) 1991 and the Swedish Panel Study of Living Conditions of the Oldest Old (SWEOLD) 2014 [15] were linked. The LNU is a nationally representative sample of people aged 18 to 75 years (79.1% response rate). LNU participants who had reached at least 70 years of age by 2014 were invited to participate in SWEOLD (84.3% response rate). Mixed interviews (0.5% of our analytical sample) and proxy interviews (3.6% of our analytical sample) were used to avoid non-response due to impaired cognition/communication or poor health. Proxies were typically a spouse, close relative, or healthcare professional.

**Figure 1.**
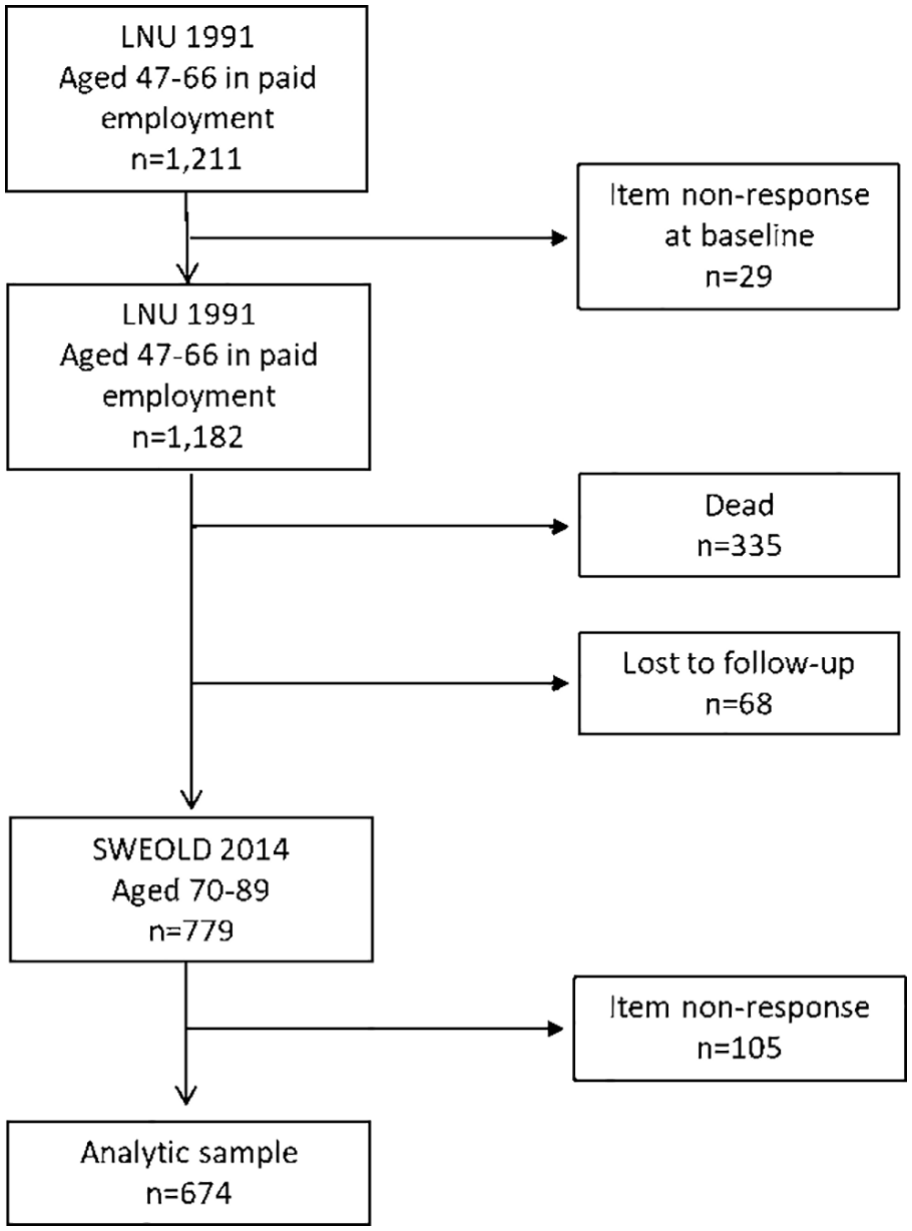
Sample size flowchart.

To minimise recall bias the questions on occupational history in LNU 1991 were only administered to participants in paid employment and aged 47–66 years (*n*=1211) [16]. Of these, 29 had item non-response at baseline, hence our baseline sample is 1182. Of these, 779 were re-interviewed for SWEOLD 2014; that is, a total of 403 respondents were lost at follow-up between 1991 and 2014 (dead, *n*=335 and other reason, *n*=68). Item non-response at follow-up (SWEOLD 2014, *n*=105) reduced the analytical sample to 674. The item non-response at follow-up was mainly because cognitive function could not be assessed through postal questionnaires (*n*=85) (see Figure 1). Our analytical sample consisted of participants aged 47–65 years at baseline (M=53.5) and 70–89 years at follow-up (M=76.5). The regional ethical review board in Stockholm approved the study protocol for SWEOLD 2014 (EPN Dnr 2014/1003-31/5) and for linking SWEOLD with LNU (EPN Dnr 2015/1070-31/5).

### Variables

#### Occupational history

We created individual trajectories of working conditions over the working life (up to eight time points). This was done by matching job exposure matrices [17, 18], which are tables with average working conditions for different occupations, with data on last recorded occupation (i.e. current occupation in LNU 1991) and occupational history recorded in LNU 1991.

Occupational history was assessed with retrospective questions about the respondent’s first occupation and all jobs thereafter. The occupations were classified using the 1980 Nordic version of the three-digit international standard occupational classification codes and were created for ‘first occupation’ (regardless of age at first job), and at ages 25, 30, 35, 40, 45 and 50 years and current occupation in 1991. About 90% of the sample had complete data on occupational history. Those 10% with missing data on occupational history had missing data on occupation at age 25 years, probably because their first job occurred after the age of 25 years.

#### Job exposure matrices: working conditions

Physically demanding, hazardous and high strain working conditions were assessed with a matrix [17] based on average scores on working conditions for 262 occupations from the 1977 and 1979 Swedish Survey of Living Conditions (ULF). In ULF, gender-specific scores were generated for physically demanding occupations (five questions), hazardous occupations (seven questions), job control (12 questions), and job demand (two questions) (Table I). All scores in the matrix consisted of a linear composite that ranged from 0 to 10. For a more comprehensive description of the matrix, see Johnson and Stewart [17]. High strain occupations were measured based on the Karasek and Theorell [19] model as the ratio of job demands to job control.

**Table I.** Description of items included to assess working conditions.

Dimension	Components
Physically demanding occupations	Unsuitable working postures Heavy lifting Work safety Physical exertion Dirt
Hazardous occupations	Noise Heavy shaking or vibrations Cold Drafts Inadequate ventilation Bad lighting Gas, mist or smoke
Job control	Planning of work Planning of vacations Planning of work breaks The selection of supervisors The selection of co-workers The setting of the work pace How time is used in work If there are varied work procedures If there is varied task content Flexible working hours Possibility to learn new things Experience of personal fulfilment on the job
Job demand	Is your job hectic? Is your job psychologically demanding?
Substantive complexity	General educational development The complexity of work with data Intellectual aptitude Numerical aptitude Verbal aptitude Temperament for repetitive and continuous processes Abstract interest in the job

The second job exposure matrix measures substantive complexity scores [18]. The scores were derived from the Dictionary of Occupational Titles (DOT) using the 1971 Current Population Survey [18]. Substantive complexity comprises eight characteristics (Table I). These items were standardised and summed to form a substantive complexity scale, which for ease of interpretation was converted to a 0–10 range. The US occupational categories have been matched with Swedish occupational categories to create substantive complexity scores for Swedish occupations.

#### Lifetime trajectories of working conditions

Trajectories of working conditions were assessed with random effects growth curve models to calculate within-person change of working conditions across working life. Growth curve models handle missing data by giving more weight to individuals with the most time points. Therefore, people with incomplete data on occupational history were included, as long as they had data on first occupation and current occupation in LNU 1991. Random effects allow for variation between participants in the individual slope and intercept. The intercept was divided into low and high via median split. The slope was divided into de-accumulation, stable and accumulation. A slope was considered as de-accumulation if there was a decrease (an estimate below zero) and as accumulation if there was an increase of more than half a standard deviation above zero. A slope between zero and half a standard deviation increase was considered a stable slope. The slope and intercept were combined to create six categories: (a) starting low and de-accumulating; (b) starting low and stable; (c) starting low and accumulating; (d) starting high and de-accumulating; (e) starting high and stable; or (f) starting high and accumulating.

#### Outcome

Successful ageing was assessed at follow-up in 2014. It was operationalised using an index of social and cultural activities (visiting friends or relatives, socialising with friends or relatives outside the home, helping family members, going to movies/theatre/concerts/museums/exhibitions during the past 12 months), cognitive function (an abridged version of the Mini-Mental State Examination) [20], physical function (self-reported ability to walk up and down stairs, standing without support, walking 100 meters fairly briskly, problems with balance indoors, and getting up from a (kitchen) chair with arms crossed over the chest), and absence of diseases (if during the past 12 months they had cancer, diabetes, stroke, depression, hypertension, heart failure and arrhythmia, or had ever had cancer, stroke, or myocardial infarction). The subcomponents of the index were z-standardised to allow for equal representation of the three domains of successful ageing, as recommended by Rowe and Kahn [13]. As the variables were not normally distributed the index was divided into quintiles. A more detailed description of the dependent variables is available in Supplemental data.

#### Covariates

Information on childhood conditions was taken from the earliest possible LNU wave; that is, 1968, 1974, 1981 or 1991, to minimise recall bias: father’s social class (blue-collar workers, including small farmers and entrepreneurs without employees; and white-collar workers, including large farmers and entrepreneurs with employees and academic professionals); father’s educational attainment (compulsory or beyond); severe conflicts within the family during childhood (yes/no or not sure); and financial difficulties during upbringing (yes/no). Level of education was collected in LNU 1991 (compulsory or beyond).

### Statistical analyses

Ordered logistic regression was used to analyse the association between working conditions and successful ageing (ordinal dependent variable). The proportional odds assumption was tested and fulfilled. A crude and an adjusted model were performed. The adjusted model included age (linear); sex (binary); father’s social class (binary); father’s educational attainment (binary); severe conflicts within the family during childhood (binary); financial difficulties during upbringing (binary); and level of education (binary). In a sensitivity analysis, we tested if there was an interaction between sex and working conditions for the different outcomes. The interaction term was only statistically significant for two out of the 40 interactions. Therefore, women and men were analysed together. All statistical analyses were conducted with STATA 15.

## Results

Participants with a higher score of successful ageing also reported better childhood conditions, had a higher level of education, were more likely to be women and on average were younger (Table II). Bivariate analyses show that, with the exception of high strain jobs, those with more advantageous lifetime working conditions had higher successful ageing scores (Table III).

**Table II.** Descriptive statistics, distribution of exposures and covariates by successful ageing, in the sample and attrition (death and non-response).

	Successful ageing^a^	Analytical sample, *n*=674	Dead at follow-up, *n*=335	Non-response, *n*=68	*P* value^b^
	Mean (SD)	*n* (%)	*n* (%)	*n* (%)	
Age					<0.001
Age 47–53	2.3 (1.3)	377 (55.9)	104 (31.0)	40 (58.8)	
Age 54–66	1.6 (1.4)	297 (44.1)	231 (69.0)	28 (41.2)	
Sex					<0.001
Women	2.1 (1.4)	349 (51.8)	107 (31.9)	27 (39.7)	
Men	1.9 (1.4)	325 (48.2)	228 (68.1)	41 (60.3)	
Level of education					<0.001
Compulsory	1.7 (1.4)	187 (27.7)	145 (43.3)	37 (54.4)	
Vocational	2.0 (1.4)	337 (50.0)	138 (41.2)	16 (23.5)	
Upper secondary	2.4 (1.4)	80 (11.9)	20 (6.0)	10 (14.7)	
University	2.6 (1.2)	70 (10.4)	32 (10.0)	5 (7.4)	
Father’s social class					0.391
Blue collar worker	1.9 (1.4)	380 (56.4)	197 (58.8)	34 (50.0)	
White collar worker	2.2 (1.3)	294 (43.6)	138 (41.2)	34 (50.0)	
Father’s education					0.016
Compulsory	2.0 (1.4)	496 (73.6)	270 (80.6)	57 (83.8)	
Beyond compulsory	2.1 (1.4)	178 (26.4)	65 (19.4)	11 (16.2)	
Financial hardship					0.039
Yes	1.7 (1.4)	123 (18.2)	70 (20.9)	21 (30.9)	
No	2.1 (1.4)	551 (81.8)	265 (79.1)	47 (69.1)	
Conflicts in childhood					0.652
Yes	1.9 (1.4)	74 (11.0)	36 (10.8)	5 (7.4)	
No	2.0 (1.4)	600 (89.0)	299 (89.3)	63 (92.7)	

a Range 0–4.

b *P* value was calculated with Pearson’s chi-square test to compare the groups (analytical sample, dead at follow-up, non-response).

**Table III.** Descriptive statistics of trajectories of working conditions by successful ageing, in the sample and attrition (death and non-response).

	Successful ageing^a^	Analytical sample, *n*=674	Dead at follow-up, *n*=335	Non-response, *n*=68	*P* value^b^
	Mean (SD)	*n* (%)	*n* (%)	*n* (%)	
Complexity					0.240
Low/de-accumulation	1.9 (1.4)	84 (12.5)	52 (15.5)	16 (23.5)	
Low/stable slope	1.8 (1.4)	92 (13.7)	37 (11.0)	11 (16.2)	
Low/accumulation	1.8 (1.4)	153 (22.7)	65 (19.4)	12 (17.7)	
High/de-accumulation	2.0 (1.4)	96 (14.2)	54 (16.1)	9 (13.2)	
High/stable slope	2.2 (1.4)	127 (18.8)	70 (20.9)	13 (19.1)	
High/accumulation	2.4 (1.3)	122 (18.1)	57 (17.0)	7 (10.3)	
High strain					<0.001
Low/de-accumulation	2.0 (1.5)	132 (19.6)	61 (18.2)	5 (7.4)	
Low/stable slope	1.9 (1.4)	78 (11.6)	38 (11.3)	5 (7.4)	
Low/accumulation	2.1 (1.4)	128 (19.0)	53 (15.8)	12 (17.7)	
High/de-accumulation	1.9 (1.4)	216 (32.1)	114 (34.0)	25 (36.8)	
High/stable slope	2.3 (1.4)	90 (13.4)	51 (15.2)	17 (25.0)	
High/accumulation	2.0 (1.4)	30 (4.5)	18 (5.4)	4 (5.9)	
Physically demanding					<0.001
Low/de-accumulation	2.3 (1.4)	250 (37.1)	86 (25.7)	11 (16.2)	
Low/stable slope	2.6 (1.3)	36 (5.3)	15 (4.5)	4 (5.9)	
Low/accumulation	2.1 (1.4)	61 (9.1)	22 (6.6)	8 (11.8)	
High/de-accumulation	1.8 (1.4)	228 (33.8)	137 (40.9)	31 (45.6)	
High/stable slope	1.7 (1.4)	46 (6.8)	50 (14.9)	10 (14.7)	
High/accumulation	1.5 (1.5)	53 (7.9)	25 (7.5)	4 (5.9)	
Hazardous					<0.001
Low/de-accumulation	2.3 (1.4)	243 (36.1)	74 (22.1)	16 (23.5)	
Low/stable slope	1.9 (1.4)	71 (10.5)	16 (4.5)	4 (5.9)	
Low/accumulation	2.0 (1.5)	31 (4.6)	9 (2.7)	3 (4.4)	
High/de-accumulation	1.9 (1.3)	266 (39.5)	183 (54.6)	39 (57.4)	
High/stable slope	2.0 (1.6)	23 (3.4)	23 (6.9)	4 (5.9)	
High/accumulation	1.6 (1.5)	40 (5.9)	30 (9.0)	2 (2.9)	

a Range 0–4.

b *P* value was calculated with Pearson’s chi-square test and Fisher’s exact test to compare the groups (analytical sample, dead at follow-up, non-response).

A high initial level of substantive complexity followed by an accumulation throughout working life was associated with higher odds of successful ageing (odds ratio (OR) 2.25, 95% confidence interval (CI) 1.34, 3.80) in the adjusted model (Table IV).

**Table IV.** Associations between working conditions across working life and successful ageing.

	Successful ageing (*n*=674)			
	Complexity	High strain	Physically demanding	Hazardous
	OR (95% CI)	OR (95% CI)	OR (95% CI)	OR (95% CI)
Trajectories across working life (Crude model)				
Low/de-accumulation	1.00	1.00	1.00	1.00
Low/stable slope	0.91 (0.54, 1.54)	1.12 (0.68, 1.84)	1.40 (0.77, 2.58)	**0.57** (0.36, 0.92)
Low/accumulation	0.93 (0.58, 1.50)	**0.62** (0.41, 0.95)	0.75 (0.46, 1.22)	0.64 (0.33, 1.28)
High/de-accumulation	1.27 (0.75, 2.13)	0.84 (0.58, 1.23)	**0.54** (0.39, 0.74)	**0.56** (0.41, 0.77)
High/stable slope	1.57 (0.96, 2.56)	1.02 (0.63, 1.64)	**0.45** (0.26, 0.80)	0.68 (0.30, 1.53)
High/accumulation	**1.86** (1.14, 3.03)	0.52 (0.26, 1.02)	**0.36** (0.21, 0.62)	**0.40** (0.22, 0.75)
Adjusted model				
Trajectories across working life (Adjusted model)				
Low/de-accumulation	1.00	1.00	1.00	1.00
Low/stable slope	0.89 (0.52, 1.51)	1.01 (0.62, 1.66)	1.28 (0.69, 2.36)	**0.56** (0.35, 0.91)
Low/accumulation	0.96 (0.59, 1.56)	**0.55** (0.36, 0.86)	0.81 (0.49, 1.34)	0.83 (0.42, 1.64)
High/de-accumulation	1.27 (0.74, 2.17)	0.87 (0.60, 1.28)	**0.62** (0.44, 0.87)	**0.67** (0.47, 0.97)
High/stable slope	1.43 (0.86, 2.40)	0.81 (0.50, 1.31)	0.56 (0.31, 1.02)	0.80 (0.34, 1.91)
High/accumulation	**2.25** (1.34, 3.80)	0.65 (0.32, 1.34)	**0.38** (0.22, 0.68)	**0.38** (0.19, 0.76)

Adjusted model: adjusted for age, sex, educational attainment and childhood conditions assessed in 1991.

Indicators of working conditions were entered into four separate models.

Results in bold *P*<0.05.

High job strain was generally not statistically significantly associated with successful ageing. The exception was participants with a low initial level of high strain and an accumulation in which we observed lower odds of successful ageing (OR 0.55, 95% CI 0.36, 0.86) in the adjusted model (Table IV).

A high starting point of physically demanding or hazardous work was associated with lower odds of successful ageing, regardless of the subsequent trajectory (Table IV). In the adjusted model, an initially high starting point followed by an accumulation and a high starting point of physically demanding work followed by a de-accumulation were both associated with lower odds of successful ageing (OR 0.38, 95% CI 0.22, 0.68 and OR 0.62, 95% CI 0.44, 0.87, respectively). Although the association between a high starting point followed by a stable trajectory and successful ageing did not reach traditional thresholds of statistical significance (i.e. *P*<0.05), it was approaching significance (Table IV). A high initial level of hazardous work followed by an accumulation was associated with lower odds of successful ageing (OR 0.38, 95% CI 0.19, 0.76) in the adjusted model (Table IV).

## Discussion

This longitudinal study investigated working conditions throughout working life in relation to successful ageing at age 70 years and beyond. In short, having higher substantive complexity at the beginning of the career, followed by an accumulation across working life, appears to be most beneficial for successful ageing. Having a high initial starting point of physically demanding or hazardous work, followed by an accumulation across working life, appears to be the least beneficial for successful ageing. Having a low initial starting point of job strain, followed by an accumulation across working life, was associated with lower levels of successful ageing.

Earlier research has shown that the effect of working conditions can reach deep into retirement [3–9]. However, the majority of these studies focus on just one health outcome. This study reveals a long-term association between both physical and psychological working conditions throughout working life and a multi-dimensional measure of successful ageing, including health, cognitive and physical function and social and leisure activities. This indicates that working life influences key aspects of later life.

The importance of taking a life course approach to predict successful ageing has been highlighted previously [11, 14, 21]. As suggested by cumulative advantage/disadvantage (CAD) theory [11] exposure to poor working conditions multiple times across working life may cause an accumulation of disadvantage with a negative influence on health later in life. Starting with a low exposure of job strain, followed by an accumulation of disadvantage, was associated with decreased chances of ageing successfully. Re-occurring experiences of job strain (chronic stress) may cause accumulated wear and tear on the body resulting in allostatic overload [22]. This may have long-term effects on the body through biochemical pathways, such as elevated levels of cortisol, which may, in turn, affect brain regions involved in cognition and memory [23]. Chronic stress may also have long-term physical impacts through physiological dysregulations that cause damage to muscles, brain, heart and vascular systems via disruptions of immune function, blood pressure, metabolism and hormone function [22]. This, in turn, increases the risk of several health conditions that pose a threat for successful ageing later in life, such as cardiovascular diseases [1]. Surprisingly, starting with a high exposure of job strain, followed by an accumulation of disadvantage, was not associated with decreased chances of ageing successfully. This is likely to be due to power issues. Still, it warrants more investigation in future research.

Those who started with a high exposure to physical or hazardous work, followed by an accumulation of disadvantage, were least likely to age successfully. High physical workload, such as heavy lifting and unsuitable working postures, has been associated with lower physical function and back pain in later life [24, 25]. Experiencing increasing levels of physical demanding and hazardous working conditions across working life may cause physical damage to the body, such as musculoskeletal pain [26] and ischaemic heart disease [1] with long-term consequences for health and function. Moreover, people with physically demanding and stressful jobs generally have poorer lifestyle habits; for example, fewer activities outside of work [19] and they smoke more [27]. They are also more likely to have lower incomes [28] and belong to a lower social class [5, 28]. This may lead to accumulated disadvantages over the life course [11].

There is a relative lack of research on ‘de-accumulation’ and ‘accumulation of advantage’ [11]. Our findings show that starting with a high exposure to physically demanding or hazardous work, compared to a low starting point, followed by a de-accumulation of disadvantage was associated with decreased chances of ageing successfully. This may indicate a change of occupation as a consequence of poor working conditions and/or poor health. Perhaps these individuals, after a long period of physically demanding or hazardous work tasks, changed their occupation, either as a result of poor health or voluntarily. Those who start their career with high substantive complexity and experience an accumulation of advantage were most likely to age successfully. Our finding of an association between intellectually stimulating jobs throughout working life and successful ageing is in line with the cognitive reserve theory [29]. This maintains that intellectually stimulating work should postpone age-related cognitive decline as the intellectually challenging occupations may function as a buffer; that is, people with high reserve can tolerate a certain degree of brain pathology. Moreover, people with good working conditions generally have healthier lifestyles; for example, greater physical, social and mental activities outside of work [19], and they do not smoke [27]. But they are also more likely to have higher incomes [10, 28] and belong to a higher social class [5, 28]. On retirement, these people may have better health and function as a result of accumulated advantage over the life course [11].

Our results should be interpreted with some caution. First, although we had a nationally representative sample with a high response rate at both baseline and follow-up, studies with the oldest old are sensitive to selective survival and attrition; that is, those with better health and life circumstances, such as working conditions, survive and continue to respond. Post hoc analyses, in which the analytical sample was compared with people who did not participate in the follow-up, confirmed this assumption (see Tables II and III). Complete responders reported better childhood circumstances, higher education and better working conditions across working life. Hence, our models are likely to underestimate the associations. However, the use of proxy interviews made it possible to represent the frailest older adults better and can partially compensate for selective non-response [30]. Second, there may also be a health selection into different types of occupations that may influence the results. We could not adjust for health before the first occupation, which would reduce the risk of reversed causality. We did, however, adjust for childhood conditions and level of education in an attempt to minimise this type of selection bias. Third, although the occupational history provided in the LNU presents a unique chance to examine working conditions across working life, this presents a risk of recall bias. To minimise such bias, all jobs held were described by the respondent one after the other in temporal order. Fourth, we were not able to adjust for other socioeconomic and lifestyle factors over the life course. Therefore, it is likely that we did not eliminate all confounders that may play a role in the relation between working conditions and successful ageing. Finally, using an average population-based matrix does not consider inter-individual variations within the same type of occupation. However, the advantage of using these matrices is that they are relatively free of bias caused by individual reporting differences and they have been shown to have good internal validity [17].

## Conclusions

To conclude, as retirement ages increase internationally, the influence of work on future health could become even more important. Understanding which working conditions contribute to successful ageing is central to the design of public health policies that aim to reduce health risks over the life course. There is a growing body of research on inequalities in health and wellbeing in later life. The majority of the research focuses on just one outcome; for example, depression, physical health, quality of life, etc. However, the different dimensions of health and wellbeing tend to be associated with one another. Hence, by using a composite measure of successful ageing we captured this multi-dimensionality. Our results suggest that policy makers, employers and practitioners should not only target isolated working conditions, or isolated health outcomes in later life, but rather take a holistic approach. The results from this study highlight the importance of improving intellectual stimulation and control, as well as reducing physically demanding, hazardous and stressful jobs throughout working life to contribute to successful ageing among people 70 years and older. Targeting modifications to working conditions early in the career might have cumulative beneficial effects for successful ageing.

## Supplemental Material

sj-docx-1-sjp-10.1177_14034948211013279 – Supplemental material for Life-course trajectories of working conditions and successful ageing Click here for additional data file. Supplemental material, sj-docx-1-sjp-10.1177_14034948211013279 for Life-course trajectories of working conditions and successful ageing by Charlotta Nilsen, Alexander Darin-Mattsson, Martin Hyde and Jonas W. Wastesson in Scandinavian Journal of Public Health
