# Supplementary material for: Life-course trajectories of working conditions and successful ageing
Source: Scand J Public Health. 2021 May 25;50(5):593–600. doi: 10.1177/14034948211013279 (PMC9203674; doi:10.1177/14034948211013279)
Supplement: sj-docx-1-sjp-10.1177_14034948211013279 – Supplemental material for Life-course trajectories of working conditions and successful ageing [file sj-docx-1-sjp-10.1177_14034948211013279.docx]

**Supplementary data. Description of variables in subdomains of the successful aging index**

**Engagement with life**

*Social activity* was assessed with five questions: “Which of the following leisure-time activities do you usually do: 1) visit friends, 2) socialize with friends outside the home, 3) visit relatives, 4) socialize with relatives outside the home, and 5) helping family members with babysitting or other small favors. Answers (no=0; yes, sometimes=1; yes, often=2). If the participant answered yes, often on one or more of the variables they were given the score of 1. *Cultural activity* was assessed with the question “Which of the following leisure time activities do you usually do? – cultural activities (going to movies, theatre, concerts, museums, exhibitions)” Answers (no=0; yes, sometimes=1; yes, often=2). If the participant answered yes sometimes or yes often, they were given the score of 1. The respondents were given the score of 1 on the ‘engagement with life’-variable if they had the score of 1 on both social and cultural activity. The respondents were given the score of 0 if they had the score of 0 on both social and cultural activity or the score of 1 on only one of them. A total of two cases were missing. Both were direct interviews (one questionnaire and one telephone interview).

**Physical and cognitive function**

*Physical function* was assessed as the self-reported ability to walk up and down stairs (yes/no), stand without support (yes/no), walk 100 meters fairly briskly (yes/no), problems with balance indoors (yes/no), and rise from a (kitchen) chair with arms crossed across the chest (yes/no). The answers were combined into an index that ranged between 0 and 5. A high score indicates good physical function. Because few people scored 0, 1, or 2, these categories were combined into one category so the final index ranged between 0 and 2 Respondents who were bedridden were given a score of 0 points. A total of two cases were missing. Both were proxy interviews (one telephone interview and one questionnaire).

*Cognitive function/communication* was assessed with an abridged version of the Mini-Mental State Examination (MMSE). The MMSE measures tasks on registration (registering and repeating three objects), orientation (year/month/date, country), delayed recall (repeat the three objects from the first task), attention/concentration (subtract 7 from 100, then keep subtracting seven from what is left; repeat five times). A high score indicates good cognitive function. The average MMSE score was 8.9 points out of a maximum of 11 (SD = 2.7). Only participants in direct interviews were given the opportunity to do the MMSE. Interviewer notes showed that proxy-interviewed persons were too sick or weak to participate directly – the majority because of cognitive problems. Therefore, respondents who did not take the test or who could not be interviewed directly, i.e. did not have the ability to communicate, were given a score of 0 points. All missing values in the variable were treated as incorrect answers. However, interviews through questionnaires (80 cases) were coded as missing.

**Absence of diseases**

*Diseases* were assessed with a 7-item index that included self-reported diabetes, arrhythmia/irregular heart rhythm, depression/deep sadness, hypertension, coronary thrombosis/myocardial infarction, malignant tumor/cancer, and cerebral thrombosis/stroke, experienced during the last 12 months. Answers were coded as “0 = severe or mild problems” or “1 = no problems”. The respondents were also asked if they ever had cancer or stroke. If they answered yes, they were given a score of 0 points. The cut-off for avoiding diseases was defined as answering no problems on all the diseases in the index. A total of 36 cases were missing. Among those 27 had direct interviews and nine mixed or proxy (22 telephone interviews and 14 questionnaires).
